# Quantitative Regulation of Interlayer Space of NH_4_V_4_O_10_ for Fast and Durable Zn^2+^ and NH_4_^+^ Storage

**DOI:** 10.1002/advs.202206836

**Published:** 2023-01-25

**Authors:** Shuyue Li, Dongxu Yu, Jingyi Liu, Nan Chen, Zexiang Shen, Gang Chen, Shiyu Yao, Fei Du

**Affiliations:** ^1^ Key Laboratory of Physics and Technology for Advanced Batteries (Ministry of Education) State Key Laboratory of Superhard Materials College of Physics Jilin University Changchun 130012 China; ^2^ Shaanxi Key Laboratory of Nanomaterials and Nanotechnology Xi'an University of Architecture and Technology Xi'an 710055 China; ^3^ Institute of Zhejiang University‐Quzhou 99 Zheda Road Quzhou Zhejiang Province 324000 China; ^4^ Division of Physics and Applied Physics School of Physical and Mathematical Sciences Nanyang Technological University Singapore 637616 Singapore

**Keywords:** ammonium vanadate, aqueous ammonium‐ion batteries, aqueous zinc‐ion batteries, interlayer space, layered structure

## Abstract

Layered vanadium‐based oxides are the promising cathode materials for aqueous zinc‐ion batteries (AZIBs). Herein, an in situ electrochemical strategy that can effectively regulate the interlayer distance of layered NH_4_V_4_O_10_ quantitatively is proposed and a close relationship between the optimal performances with interlayer space is revealed. Specifically, via increasing the cutoff voltage from 1.4, 1.6 to 1.8 V, the interlayer space of NH_4_V_4_O_10_ can be well‐controlled and enlarged to 10.21, 11.86, and 12.08 Å, respectively, much larger than the pristine one (9.5 Å). Among them, the cathode being charging to 1.6 V (NH_4_V_4_O_10_‐C1.6), demonstrates the best Zn^2+^ storage performances including high capacity of 223 mA h g^−1^ at 10 A g^−1^ and long‐term stability with capacity retention of 97.5% over 1000 cycles. Such superior performances can be attributed to a good balance among active redox sites, charge transfer kinetics, and crystal structure stability, enabled by careful control of the interlayer space. Moreover, NH_4_V_4_O_10_‐C1.6 delivers NH_4_^+^ storage performances whose capacity reaches 296 mA h g^−1^ at 0.1 A g^−1^ and lifespan lasts over 3000 cycles at 5 A g^−1^. This study provides new insights into understand the limitation of interlayer space for ion storage in aqueous media and guides exploration of high‐performance cathode materials.

## Graphical abstract

Here, it is shown that via increasing the cutoff voltage from 1.4, 1.6, to 1.8 V, the interlayer space of NH_4_V_4_O_10_ can be well‐controlled and enlarged quantitatively to 10.21, 11.86, and 12.08 Å, respectively, much larger than the pristine one (9.5 Å), resulting in better zinc‐ion storage performance.

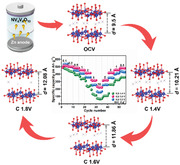

## 1 Introduction

Aqueous zinc‐ion batteries (AZIBs) have shown great promise in the large‐scale energy storage owing to their intrinsic features of using water as the electrolyte which possesses high ionic conductivity, low cost, and safety over its flammable organic counterpart. Furthermore, AZIBs could use Zinc metal as the anode directly which is expected to deliver a high energy density because of its high theoretical capacity of 820 mA h g^−1^ and low redox potential of −0.76 V (vs standard hydrogen electrode).^[^ ^1^, ^2^, ^3^ ^]^ Among potential cathode materials for Zn^2+^ ions storage, layered vanadium‐based oxides have attracted wide attention because of their unique 2D layered structure and multiple redox reactions from V^2+^ to V^5+^ enabling plenty of active sites for Zn^2+^ storage.^[^ ^4^, ^5^ ^]^ However, the strong electrostatic interaction between Zn^2+^ and the lattice framework usually results in the sluggish diffusion kinetics and structural instability with the result of unsatisfied cycle lives and rate capability.^[^ ^6^, ^7^, ^8^ ^]^

So far, a feasible strategy to improve Zn^2+^ storage performance is introducing alien ions or molecules into the interspaces of V—O layers, which works as the pillars to enlarge the cation migration paths, weaken the electrostatic interaction, and create more active sites, therefore accelerate Zn^2+^ diffusion kinetics. Furthermore, the pillars could hold up the layer structure during a large amount of Zn^2+^ extraction, avoiding the structural collapse, thus guarantee long‐term stability.^[^ ^9^, ^10^ ^]^ For examples, Kundu et al. proposed water molecules preintercalated Zn_0.25_V_2_O_5_·nH_2_O cathode with an improved capacity retention of over 80% after 1000 cycles.^[^ ^11^ ^]^ Yang et al. synthesized Li*_x_* V_2_O_5_·nH_2_O by chemical intercalation of Li^+^ into the interlayer of V_2_O_5_·nH2O, which exhibited stable cycling performance of 192 mAh g^−1^ after 1000 cycles at 10 A g^−1^.^[^ ^12^ ^]^ Bin et al. successfully introduced conducting poly(3,4‐ethylenedioxythiophene) (PEDOT) into NH_4_V_3_O_8_ to expand the interlayer spacing from 7.8 to 10.8 Å with promoted rate capability of 163 mAh g^−1^ at 10 A g^−1^.^[^ ^13^ ^]^ Despite the achievements obtained, there is lack of the in‐depth understanding between the improved electrochemical performance and the widening interlayer space. To answer whether there is a limitation of space for ion storage, it is of importance to regulate the interlayer distance quantitatively, whereas it is hard to realize it using the ordinary synthesis strategies.

**Figure 1:**
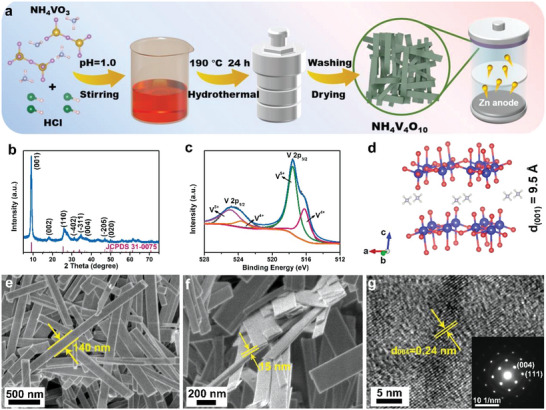
a) Schematic illustration of the preparation procedure for NH_4_V_4_O_10_ nanoribbon. b) X‐ray diffraction pattern of the as‐prepared NH_4_V_4_O_10_. c) V 2p XPS spectrum of the as‐prepared NH_4_V_4_O_10_. d) Schematic crystal structure of the NH_4_V_4_O_10_. e,f) SEM, and g) HRTEM images of NH_4_V_4_O_10_ nanoribbon with SAED in the inset.

Herein, we propose an in situ electrochemical strategy to engineer the interlayer space of NH_4_V_4_O_10_ quantitatively, which reveals a close relationship between the optimal electrochemical performance with interlayer space. The reason for selecting NH_4_V_4_O_10_ as the model could be attributed to its layered structure, where NH_4_^+^ ions work as the pillar and are located as the interspace of V—O layers as shown in **Figure** **1**d.^[^ ^14^ ^]^ More importantly, it has been demonstrated that NH_4_^+^ ions could be reversibly extracted and inserted in the water‐media, which enables an opportunity to accurately control the interlayer space via regulating the extraction content of NH_4_^+^ ions.^[^ ^15^ ^]^ As expected, via increasing the upper cutoff voltage from 1.4, 1.6, to 1.8 V, different amount of NH_4_^+^ could be extracted from NH_4_V_4_O_10_, with different well‐controlled interlayer distances of 10.21, 11.86, and 12.08 Å, respectively. Surprisingly, the deammoniated material with the interlayer spacing of 11.86 Å exhibits the best performances, including high capacity of 521 mA h g^−1^ at 0.1 A g^−1^ and 223 mA h g^−1^ even at 10 A g^−1^ among the best rate performances in the state‐of‐the‐art vanadium‐based cathodes for AZIBs. Combination measurements of X‐ray diffraction (XRD) and scanning electron microscopy (SEM) reveal the deammoniated sample with space of 11.86 Å exhibits the best structural stability after long‐term cycling, which illustrates the vital role of quantitative regulation for interlayer space. In addition, NH_4_V_4_O_10_ demonstrates excellent NH_4_^+^ storage performances which exhibits a high reversible capacity of 296 mAh g^−1^ at 0.1 A g^−1^ and superior rate capability of 90 mAh g^−1^ at 5 A g^−1^, further demonstrating the superiority of designing layered cathodes with appropriate space for aqueous batteries.

## 2 Results and Discussion

### 2.1 Physical and Morphological Properties of NH_4_V_4_O_10_

Nanoribbon‐like NH_4_V_4_O_10_ is successfully prepared via one‐step hydrothermal method as schematically illustrated in Figure 1a and the detail synthesis procedure is presented in the Experimental Section. Its crystal structure was characterized via XRD, as shown in Figure 1b. All the diffraction peaks are readily assigned to a pure monoclinic structure of NH_4_V_4_O_10_ (JCPDS Card No. 31‐0075) with space group of C2/m. The intensity of (001) peak is extremely high, which illustrates the preferential growth of the material along the *c* axis.^[^ ^16^ ^]^ The Fourier transform infrared (FTIR) spectrum (Figure S1, Supporting Information) suggests a series of absorption bonds in the detected region: the three (534.5, 752.9, and 999.1 cm^−1^) in the lower bands are assigned to V—O bending, V—O—V, and V=O stretching vibrations respectively, the 1407.8 and 3185.1 cm^−1^ are owing to the bending and stretching vibrations of N—H bonds, the 1636.3 cm^−1^ can be ascribed to O—H bending.^[^ ^17^ ^]^ X‐ray photoelectron spectroscopy (XPS) was then carried out to identify the elemental composition and chemical states of NH_4_V_4_O_10_, as presented in Figure S2 (Supporting Information); and Figure 1c. The V 2p high‐resolution XPS spectrum can be deconvoluted into the overlapped V^5+^ (2p_3/2_: 517.5 eV) and V^4+^ (2p_3/2_: 516.3 eV) peaks to keep charge neutrality, in consistence with the previous reports.^[^ ^18^ ^]^ SEM (Figure 1e,f) and transmission electron microscopy (TEM) images (Figure S3, Supporting Information) show that NH_4_V_4_O_10_ crystallizes into uniform nanoribbon morphology with 140 nm in width and several micrometers in length. The lateral view of a nanoribbon (Figure 1f) suggests the thickness is about 15 nm. High‐resolution transmission electron microscopy (HRTEM) (Figure 1g) suggests that the adjacent lattice plane is 0.24 nm, in good agreement with the (004) plane of NH_4_V_4_O_10_ crystal. The corresponding selected‐area electron diffraction (SAED), as displayed in the inset of Figure 1g, reveals a series of well‐defined diffraction spots, indicative of highly crystalline NH_4_V_4_O_10_.

### 2.2 Zn^2+^ Storage Properties at Different Voltage Regions

**Figure 2:**
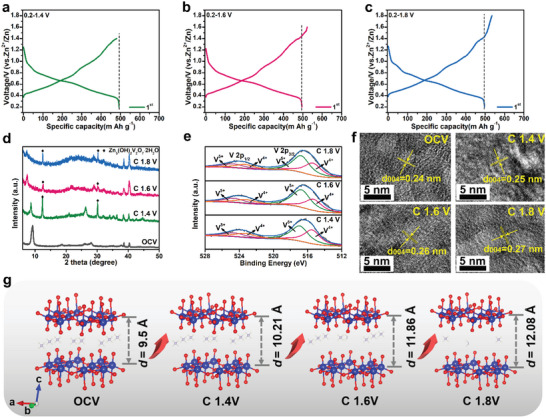
The initial galvanostatic charge and discharge profiles of NH_4_V_4_O_10_ in the voltage range of a) 0.2–1.4 V, b) 0.2–1.6 V, c) 0.2–1.8 V. d) Ex situ XRD, e) XPS, and f) HRTEM patterns of the NH_4_V_4_O_10_ electrode at different state of charge. g) Schematic crystal structure patterns of the electrode at different state of charge.

The electrochemical properties of NH_4_V_4_O_10_ as the cathode material for AZIBs is evaluated by assembling the coin‐type cell using 3 m Zn(CF_3_SO_3_)_2_ electrolyte. It is worth noting that the electrochemical processes were carried out in the order of discharging to 0.2 V first and then charging to 1.4, 1.6, and 1.8 V, respectively. As compared in **Figure** **2**a–c, NH_4_V_4_O_10_ delivers the identical initial discharge capacity when the low cutoff voltages are set as 0.2 V, indicative of capability of Zn^2+^ insertion into NH_4_V_4_O_10_ lattice. While, with increasing the upper cutting voltages from 1.4 (hereafter denoted as NH_4_V_4_O_10_‐C1.4), 1.6 (NH_4_V_4_O_10_‐C1.6) to 1.8 (NH_4_V_4_O_10_‐C1.8) V, different charging capacities are achieved as 486, 521, and 531 mAh g^−1^, respectively, accompanied by different initial Coulombic efficiency (ICE) of 98%, 105%, and 107%. The overhigh ICE above 100% suggests part NH_4_^+^ ions could be extracted from the layered structure with the result of different interlayer spacings. Then, XRD patterns of different samples were collected after experiencing initial discharging and charging process, as shown in Figure 2d. In comparison with the XRD patterns at the open‐circuit‐voltage (OCV), several new peaks appear which can indexed by the normal byproduct Zn_3_(OH)_2_V_2_O_7_·2H_2_O.^[^ ^19^ ^]^ Moreover, worthy to note that the (001) peak shifts to its low‐angle region as the charging voltage increases from 1.4 to 1.8 V. Correspondingly, the *c*‐value increase from 9.5 Å at the OCV to 10.21 Å (NH_4_V_4_O_10_‐C1.4), 11.86 Å (NH_4_V_4_O_10_‐C1.6) to 12.08 Å (NH_4_V_4_O_10_‐C1.8) calculated via the least‐square method. The increase in the *c*‐value can be understood in terms of the increasing repulsive force between the nearest O^2−^ after part NH_4_^+^ ions, which is normal in the layered cathode materials in lithium‐ion or sodium‐ion batteries.^[^ ^20^, ^21^ ^]^ Additionally, the peak intensity of NH_4_V_4_O_10_‐C1.8 significantly weakened, which might be ascribed to the excessive NH_4_^+^ vacancies cause deterioration of the crystallinity of NH_4_V_4_O_10_. In addition, HRTEM measurements (Figure 2f) show a continuous expansion of the interplanar distance along (004) which increases from 0.24 nm at OCV to 0.27 nm after charging to 1.8 V. Besides the change in the *c*‐value, the ratio of V^5+^/V^4+^ content increase accordingly since more NH_4_^+^ ions are extracted from the lattice. As shown in Figure 2e, the peaks at 516.9 and 515.2 eV are assigned to the V 2p_2/3_ of V^5+^ and V^4+^, respectively, and the ratio of V^5+^/V^4+^ content of NH_4_V_4_O_10_‐C1.8 is calculated as 1.7, higher than the value of 1.4 (NH_4_V_4_O_10_‐C1.6) and 1.2 (NH_4_V_4_O_10_‐C1.4). In sum, the change in the crystal structure and interlayer space of NH_4_V_4_O_10_ at different stages of charging are schematically shown in Figure 2g, which not only suggests the deammoniation process could enhance the interlayer space quantitatively, but also enables a unique opportunity to investigate its effect on the electrochemical performances.

**Figure 3:**
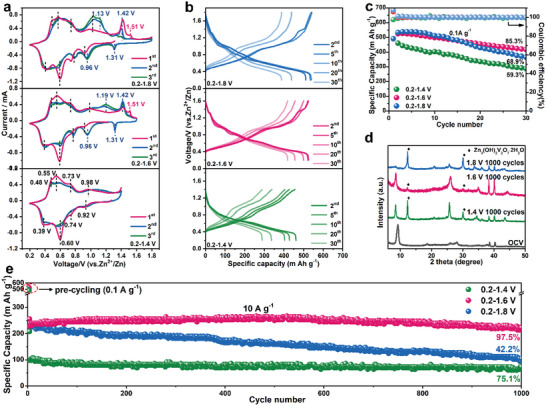
a) CV profiles of NH_4_V_4_O_10_ in the voltage range of 0.2–1.4,1.6, and 1.8 V at a scan rate of 0.2 mV s^−1^. b) Galvanostatic charge and discharge profiles of NH_4_V_4_O_10_ in the voltage range of 0.2–1.4, 1.6, and 1.8 V at a current density of 0.1 A g^−1^. c) Cycle performance of NH_4_V_4_O_10_ in 0.2–1.4, 1.6, and 1.8 V at 0.1 A g^−1^. d) Ex situ XRD of the NH_4_V_4_O_10_ electrode between different voltage range after 1000 cycles. e) Long cycle performance of NH_4_V_4_O_10_ in 0.2–1.4, 1.6, and 1.8 V at 10 A g^−1^ after pre‐cycling for three cycles at 0.1 A g^−1^.

Cyclic voltammograms (CV) profiles were then employed to study the Zn^2+^ storage properties of the samples charged to different voltage regions. As displayed in **Figure** **3**a, there are four pairs of redox peaks located at 0.92/0.98, 0.74/0.73, 0.60/0.55, and 0.39/0.48 V during the initial cycle for NH_4_V_4_O_10_‐C1.4, which suggests a multistep reaction consistent with the previous reports.^[^ ^22^, ^23^ ^]^ In contrast, when charging the cell to 1.6 and 1.8 V, a new redox peak appears at 1.51 V in the initial anodic scanning and disappears subsequently. This interesting behavior could be attributed to the irreversible extraction behavior of NH_4_^+^ ions from the layered structure. Correspondingly, two additional pairs of redox peaks appear at 1.19/0.96 and 1.42/1.31 V, possibly related to the improved Zn^2+^ storage sites after NH_4_^+^ ions’ extraction. After experiencing the initial irreversible de‐ammoniation, NH_4_V_4_O_10_ delivers a higher discharge capacity of 521 (NH_4_V_4_O_10_‐C1.6) and 531 mA h g^−1^ (NH_4_V_4_O_10_‐C1.8), as compared with value of NH_4_V_4_O_10_‐C1.4, with nearly 100% CE (Figure 3b). After 30 cycles, the electrode of NH_4_V_4_O_10_‐C1.6 still delivers a high capacity of 420 mA h g^−1^ with high‐capacity retention of 85.3%, much better than the retention of NH_4_V_4_O_10_‐C1.4 (59.3%) and NH_4_V_4_O_10_‐C1.8 (68.9%) (Figure 3c). The difference is much obvious at high applied current densities, as displayed in Figure 3e. At applied current of 10 A g^−1^, the electrode of NH_4_V_4_O_10_‐C1.6 demonstrate the best long‐term stability with a capacity retention of 97.5% after 1000 cycles, much higher than NH_4_V_4_O_10_‐C1.4 (75.1%) and NH_4_V_4_O_10_‐C1.8 (42.2%). To explain the underlying mechanism for the improved cycle stability, the long‐term structural and micro‐morphological properties of different samples after 1000 cycles were investigated. As shown in Figure 3d, NH_4_V_4_O_10_‐C1.8 loses its original layered structure, where all the diffraction peaks disappear accompanied by the transformation to Zn_3_(OH)_2_V_2_O_7_·2H_2_O. Correspondingly, its morphology (Figure S4, Supporting Information) is destroyed. Recently, the formation of the Zn_3_(OH)_2_V_2_O_7_·2H_2_O byproduct within the aqueous electrolyte containing Zn^2+^ was reported to be associated with the dissolution of vanadium. It is worth noting that the Zn_3_(OH)_2_V_2_O_7_·2H_2_O shows no electrochemical activity and blocks the ion transport during the charge/discharge process, which could lead to inferior cycle performance.^[^ ^24^ ^]^ In contrast, the NH_4_V_4_O_10_‐C1.6 and NH_4_V_4_O_10_‐C1.4 electrodes maintain their structural (Figure 3d) and morphological integrity (Figures S5 and S6, Supporting Information). Though the increasing interlayer space is beneficial to the enhanced capacity, the excessive extraction would unavoidably result in the poor structural stability, sluggish rate capability and unsatisfied cycle performance. Therefore, quantitative regulation of the interlayer space is critical for achieving optimal electrochemical performance of layered vanadium oxides.

**Figure 4:**
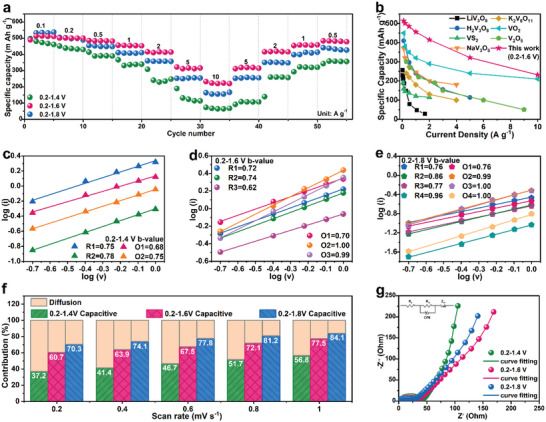
a) Rate performance of NH_4_V_4_O_10_ in the voltage range of 0.2–1.4, 1.6, and 1.8 V. b) The rate performance comparison of NH_4_V_4_O_10_ in 0.2–1.6 V versus state‐of‐the‐art materials.^[^ ^25^, ^26^, ^27^, ^28^, ^29^, ^30^, ^31^ ^]^ The *b*‐values of NH_4_V_4_O_10_ electrode between c) 0.2 and 1.4 V, d) 0.2 and 1.6 V, e) 0.2 and 1.8 V. f) The capacity contribution of NH_4_V_4_O_10_ at different scan rates between the three voltage ranges. g) Nyquist plots of the impedance spectra of NH_4_V_4_O_10_ electrode at different charge state.

Besides the enhanced cycle performance, the deammoniated materials also demonstrate an improved rate capability. As shown in **Figure** **4**a, the electrode of NH_4_V_4_O_10_‐C1.8 renders the highest reversible capacity among the three samples at 0.1 A g^−1^. With increasing the current densities, the NH_4_V_4_O_10_‐C1.6 renders the best rate performances. At 10 A g^−1^, the electrode delivers the highest reversible discharge capacity of 223 mA h g^−1^, superior to NH_4_V_4_O_10_‐C1.8 (156 mA h g^−1^) and NH_4_V_4_O_10_‐C1.4 (64 mA h g^−1^). As compared in Figure 4b, NH_4_V_4_O_10_‐C1.6 shows remarkable competitive advantages among the‐state‐of‐the‐art vanadium‐based Zn‐storage materials, especially at high current densities.^[^ ^25^, ^26^, ^27^, ^28^, ^29^, ^30^, ^31^ ^]^ Due to the poor structural stability and sluggish kinetic properties, various layered‐structure vanadium‐based compounds, such as V_2_O_5_ and VS_2_ could only deliver the capacity of 50 and 115.5 mA h g^−1^ at 9 and 2 A g^−1^, respectively, which are significantly lower than that of NH_4_V_4_O_10_‐C1.6 (223 mA h g^−1^ at 10 A g^−1^).^[^ ^29^, ^30^ ^]^ Significantly, compared with other reported NH_4_V_4_O_10_ with cation deficiency, which are synthesized by heat treatment or introducing alien ions, NH_4_V_4_O_10_‐C1.6 obtained by in situ electrochemical process is easy to operate and could cause controllable defect sites and concentration.^[^ ^32^, ^33^, ^34^, ^35^ ^]^

To reveal the underlying mechanism for the superior rate capability, the CV profiles at different scan rates from 0.2 to 1.0 mV s^−1^ (DSCV) were measured to investigate the kinetic property, as shown in Figures S7–S9 (Supporting Information). Obviously, the shape of CV curves is well preserved at increased scan rates in the three voltage ranges, demonstrating the quick response of NH_4_V_4_O_10_ electrode at high current densities. As for the electrochemical energy storage, several processes could contribute to the total stored charge, including the faradaic contribution from the diffusion‐controlled charge storage and the surface charge transfer, and the non‐faradaic contribution of double layer capacitance. Based on the previous reports, the current response for surface capacitive contribution (*i*_C_) is proportional to the scan rate (*v*), whereas the current related to the diffusion‐controlled procedure (*i*_D_) is proportional to the square root of the scan rate. Accordingly, the charge storage mechanism can be qualitatively deduced by Equation (1)^[^ ^36^ ^]^

(1) $$i=av^{b}$$

where *a* and *b* are two various numbers. A *b*‐value of 1 represents the capacitance dominated process, while it tends to be 0.5 for the diffusion‐controlled process. For NH_4_V_4_O_10_‐C1.4, the *b*‐values are 0.68 and 0.75 for cathodic peaks, as well as 0.75 and 0.78 for anodic peaks (Figure 4c), suggesting a combination of surface charge storage process with the diffusion‐controlled charge storage process. When increasing the upper charging voltage to 1.6 and 1.8 V, the calculated *b*‐values are significantly improved and approach close to 1 (Figure 4d,e), which means the a dominant pseudocapacitive contribution during reversible charge and discharge processes. Quantitatively, the scanning rate dependent current *i*(*v*) can be separated into pseudocapacitive *k*_1_ *v* and diffusion‐controlled *k*_2_ *v* ^1/2^ contributions via Equation (2)

(2) $$i\left(v\right)=k_{1}v+k_{2}v^{1/2}$$

As the sweep rate increases, the capacitive contribution significantly increases from 56.8% to 84.1% (Figure 4f) at 1 mV s^−1^. The pseudocapacitive charge storage mechanism could be established for NH_4_V_4_O_10_ with larger interlayer spacing that enables superior rate energy storage rather than that with interlayer spacing of 10.21 Å for NH_4_V_4_O_10_‐C1.4. Electrochemical impedance spectroscopy (EIS) measurements (Figure 4g) were conducted to study the reaction kinetics of the electrode material at different state during charging process. As shown in the equivalent circuit (inset of Figure 4g), *R*_s_ represents all equivalent series resistance while *R*_ct_ indicates the charge transfer resistance between the electrode/electrolyte interface. In comparison with NH_4_V_4_O_10_‐C1.4, *R*_ct_ decreases to 26 from 36 Ω (Table S1, Supporting Information). The reduced charge transfer impedance will increase the electrode kinetics and consequently improve the rate performance.^[^ ^37^, ^38^ ^]^ Although the increase of interlayer space effectively improves the kinetic properties of NH_4_V_4_O_10_ for Zn^2+^ storage, the excessively NH_4_^+^ extraction could result in the collapse of the crystal structure.

### 2.3 NH_4_^+^ Storage Properties

**Figure 5:**
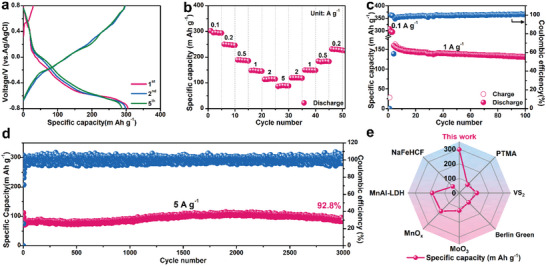
Electrochemical performance of NH_4_V_4_O_10_ in 5 m (NH_4_)_2_SO_4_ electrolyte: a) GCD curves at the current density of 0.1 A g^−1^. b) Rate capability of NH_4_V_4_O_10_ at a current density from 0.1 to 5 A g^−1^. c) Cycle performance at 1 A g^−1^. d) Long cycle performance at 5 A g^−1^. e) Comparison of NH_4_V_4_O_10_ versus state‐of‐the‐art materials for NH_4_^+^ storage in the current density of 0.1 A g^−1^.^[^ ^39^, ^40^, ^41^, ^42^, ^43^, ^44^, ^45^ ^]^

Since NH_4_^+^ ions can be extracted from the NH_4_V_4_O_10_ lattice, we further investigate the NH_4_^+^ storage properties of NH_4_V_4_O_10_ as the cathode for aqueous ammonium ion batteries (AAIBs). **Figure** **5**a presents the GCD profiles of NH_4_V_4_O_10_ at 0.1 A g^−1^. Upon initial charging to 0.8 V (vs Ag/AgCl), a small amount of NH_4_^+^ could be extracted from the crystal lattice with a charging capacity of 28 mA h g^−1^, close to the extraction content in aqueous zinc ion batteries (Figure 2b). Subsequently, NH_4_V_4_O_10_ delivers a discharge capacity of 305 mA h g^−1^ at a current density of 0.1 A g^−1^, which is the highest value among the state‐of‐the‐art materials for NH_4_^+^ ion storage owing to the 2D layered structure and multiple redox reactions of vanadium (Figure 5e; and Table S2, Supporting Information).^[^ ^39^, ^40^, ^41^, ^42^, ^43^, ^44^, ^45^ ^]^ Additionally, at high applied current density of 5 A g^−1^, NH_4_V_4_O_10_ can deliver a specific capacity of 90 mA h g^−1^ which recovers to 230 mA h g^−1^ when the current density goes back to 0.2 A g^−1^, demonstrating a superior rate capability in aqueous NH_4_^+^ storage (Figure 5b). The cycle performance of NH_4_V_4_O_10_ was also recorded at current density of 1 A g^−1^ (Figure 5c). After initial activation process, a specific capacity of 154 mA h g^−1^ can be delivered with a capacity retention of 74% over 100 cycles with high Coulombic efficiency of nearly 100%. Remarkably, NH_4_V_4_O_10_ could sustain its performance over 3000 cycles at high current density of 5 A g^−1^ with capacity retention of 92.8% (Figure 5d). In order to explore the origin of high rate capability of NH_4_^+^ storage, the CV curves at different scanning rates was measured to investigate its kinetic behaviors (Figure S10, Supporting Information). The corresponding *b*‐values of the three pairs of redox peaks are 0.62, 0.79, 1.00, 0.61, 0.85, and 0.72 (Figure S11, Supporting Information), respectively. In addition, the capacitive contribution at 1.0 mV s^−1^ is calculated as 55.8% (Figure S12, Supporting Information), suggesting the combination of surface charge storage process with the diffusion‐controlled charge storage process.

**Figure 6:**
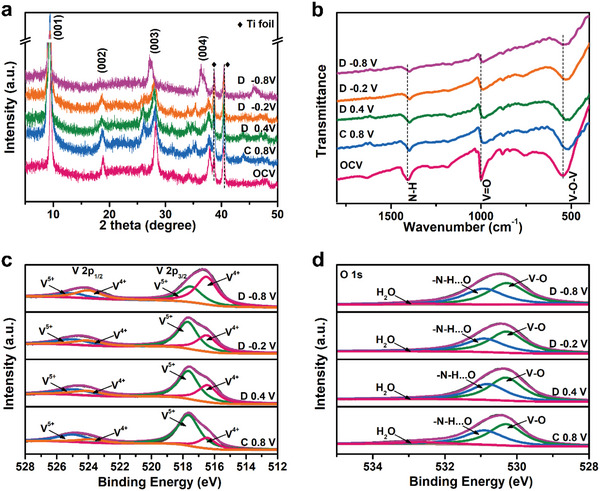
Ex situ a) XRD. b) FTIR patterns of NH_4_V_4_O_10_ in NH_4_^+^ battery. c) V 2p and d) O 1s of ex situ XPS spectrums of NH_4_V_4_O_10_ in NH_4_^+^ battery.

To explain the NH_4_^+^ storage mechanism in NH_4_V_4_O_10_, ex situ XRD, FTIR, and XPS at different charge–discharge stages were conducted. As displayed in **Figure** **6**a, during the initial charging to 0.8 V, (001) and (004) peaks shift to their lower angle side, indicative of enlarged *c*‐value, similar to the Zn^2+^ extraction behavior in Figure 2d. Upon discharging process, NH_4_V_4_O_10_ shows a solid‐solution process with no sign of forming new phases, which suggests NH_4_^+^ can be successfully intercalated into the interspacing of layers. The FTIR spectra are also utilized to analyze the chemical bonding during electrochemical process (Figure 6b). The peak featured as the vibration of V—O—V bond (534.5 cm^−1^) exhibits a redshift upon charging to 0.8 V, means the elongation of V—O—V bond, which is consistent with the expanded interlayer space. XPS of V 2p spectrum (Figure 6c) also confirms that with the intercalation of NH_4_^+^, most of V^5+^ are reduced to V^4+^. The existence of V^5+^ could due to the incomplete redox reaction results that the delivered specific capacity is lower than theoretical capacity. The O 1s spectrum (Figure 6d) can be deconvoluted into two strong peaks centered at 530.2 and 530.9 eV, corresponding to V—O and N—H⋯O hydrogen in the interlayer, respectively, which reveals that the NH_4_^+^ inserted into NH_4_V_4_O_10_ lattice.^[^ ^40^, ^46^ ^]^ The H_2_O peak located at 532.4 eV is almost undetectable during the reduction process, demonstrating only NH_4_^+^ is inserted and extracted rather than proton and hydronium ion as charge carrier.

## 3 Conclusion

In summary, an in situ electrochemical induced quantitatively interlayer space regulation for layered‐structure NH_4_V_4_O_10_, for the first time, was applied in aqueous zinc ion and ammonium ion batteries. The interlayer space of NH_4_V_4_O_10_ can be successfully expanded from 9.5 to 11.86 Å due to the partial extraction of NH_4_^+^ from the lattice accompanied by the oxidation from V^4+^ to V^5+^, which could effectively facilitate the accommodation for Zn^2+^, improve the stability of the electrode and enable quicker reaction kinetics. Moreover, NH_4_V_4_O_10_ also delivers superior NH_4_^+^ storage capability, which exceeds the reported materials before. The in situ electrochemical induced larger interlayer space cathodes provide a convenient and efficient strategy to design high performance materials for aqueous ion batteries.

## 4 Experimental Section

### Synthesis of NH_4_V_4_O_10_ Nanoribbons

NH_4_VO_3_ was purchased from Sigma‐Aldrich and used without purification. NH_4_V_4_O_10_ nanoribbons were prepared via a hydrothermal route. NH_4_VO_3_ (0.6 g) was dissolved in 150 mL of the mixture of deionized water and ethanol (9:1 in the volume ratio), and then dilute HCl aqueous solution was added to the above solution to adjust the pH value to 1.0. The mixture was stirred for 30 min. Subsequently, the mixture was subjected to hydrothermal treatment at 190 °C for 24 h and allowed to cool naturally. The final products were washed by distilled water and ethanol several times and dried at 80 °C overnight to obtain pure NH_4_V_4_O_10_ nanoribbons. The as‐prepared NH_4_V_4_O_10_ was taken directly for further characterizations.

### Material Characterization

XRD patterns were obtained on a Smartlab SE with Cu K*α* radiation. The morphologies were analyzed by the field emission SEM (Regulus 8100). TEM, HRTEM, and SAED were investigated using an FEI Tecnai G2 F20 S‐TWIN. FTIR spectra of the experimental products were recorded on a Bruker VERTEX 70 FTIR spectrometer. The valence states of the samples were identified by XPS by using a VG scientific ESCALAB‐250 spectrometer.

### Electrochemical Characterization

The working electrodes were prepared by rolling a mixture on a Ti foil current conductor, which was prepared by mixing the active material NH_4_V_4_O_10_, Super P conductive additive, and polyvinylidene fluoride (PVDF) binder at a weight ratio of 7: 2: 1. The electrode films were dried in a vacuum oven at 80 °C for 12 h and divided into small plates with a loading mass of 1.0–1.2 mg cm^−2^. 2032‐type coin cells were fabricated with Zn foil as the counter electrode, glass fiber filter (Whatman GF/C) as the separator, and 3.0 m Zn(CF_3_SO_3_)_2_ as the electrolyte. The electrochemical measurements in ANIBs were performed with Swagelok cells with activated carbon as the counter electrode, the Ag/AgCl as the reference electrode and 5.0 m (NH_4_)_2_SO_4_ as the electrolyte, respectively. All the electrochemical tests were carried out at room temperature. The galvanostatic charge/discharge performance was performed on a Land‐2001A (Wuhan, China) automatic battery tester. CV and EIS were performed on a VSP multichannel potentiostatic‐galvanostatic system (Bio‐Logic SAS, France).

## Conflict of Interest

The authors declare no conflict of interest.

## Supporting information

Supporting Information

## Data Availability

The data that support the findings of this study are available from the corresponding author upon reasonable request.
