# Supplementary material for: Quantitative Regulation of Interlayer Space of NH4V4O10 for Fast and Durable Zn2+ and NH4 + Storage
Source: Adv Sci (Weinh). 2023 Jan 25;10(9):2206836. doi: 10.1002/advs.202206836 (PMC10037961; doi:10.1002/advs.202206836)
Supplement: Supplementary file 1 — Supporting Information [file ADVS-10-2206836-s001.pdf]

Supporting Information

Quantitative Regulation of Interlayer Space of $NH_4V_4O_{10}$ for Fast and Durable Zn^{2+} and NH_4^+ Storage

Shuyue Li, Dongxu Yu, Jingyi Liu, Nan Chen, Zexiang Shen, Gang Chen, Shiyu Yao, and Fei Du**

Dr. S. Li, Dr. D.Yu, J. Liu, Dr. N. Chen, Prof. G. Chen, Prof. S. Yao, Prof. F. Du
Key Laboratory of Physics and Technology for Advanced Batteries (Ministry of Education), State Key Laboratory of Superhard Materials, College of Physics, Jilin University, Changchun, 130012, China
E-mail: yaoshiyu@jlu.edu.cn (Prof. S. Yao); dufei@jlu.edu.cn (Prof. F. Du)

Dr. S. Li
Shaanxi Key Laboratory of Nanomaterials and Nanotechnology, Xi'an University of Architecture and Technology, Xi'an, 710055, China

Dr. D.Yu
Institute of Zhejiang University-Quzhou, 99 Zheda Road, Quzhou, Zhejiang Province, 324000, China

Prof. Z. Shen
Division of Physics and Applied Physics, School of Physical and Mathematical Sciences, Nanyang Technological University, Singapore 637616, Singapore

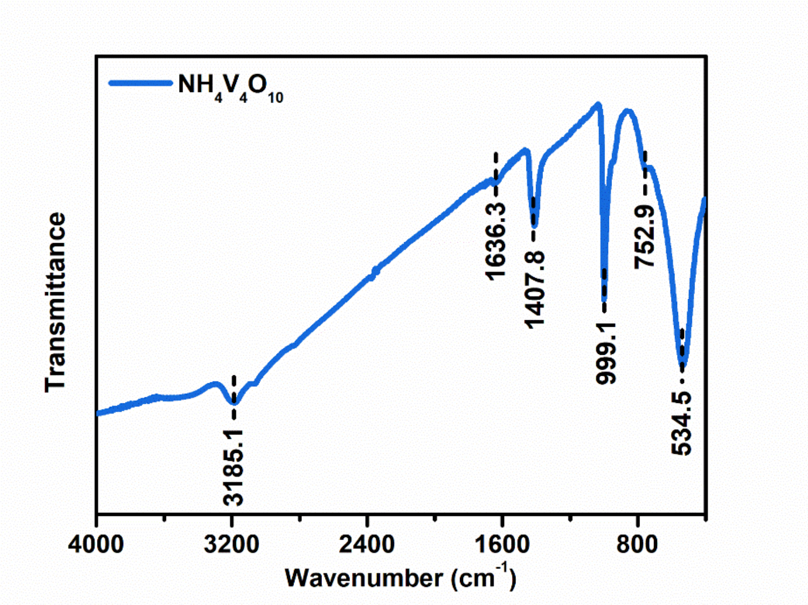

Figure S1. FTIR spectrum of $NH_4V_4O_{10}$.

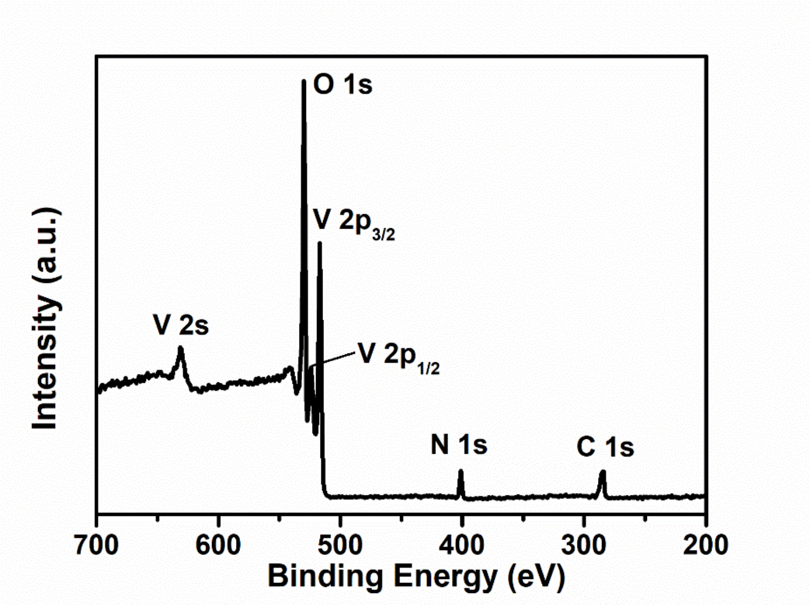

Figure S2. Survey XPS spectrum of $NH_4V_4O_{10}$.

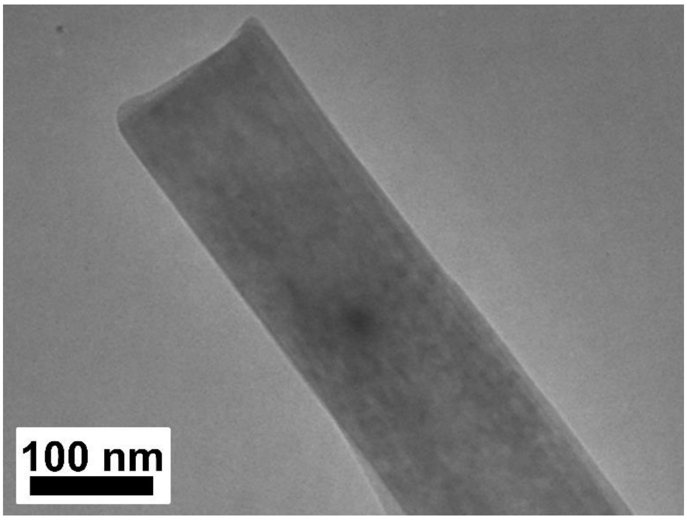

Figure S3. TEM image of $NH_4V_4O_{10}$ nanoribbon.

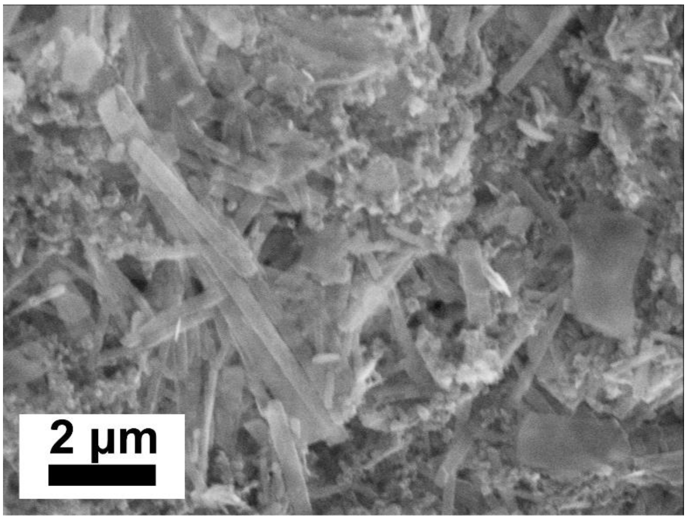

Figure S4. SEM image of $NH_4V_4O_{10}$ electrode after 1000 cycles in the voltage range of 0.2-1.8 V.

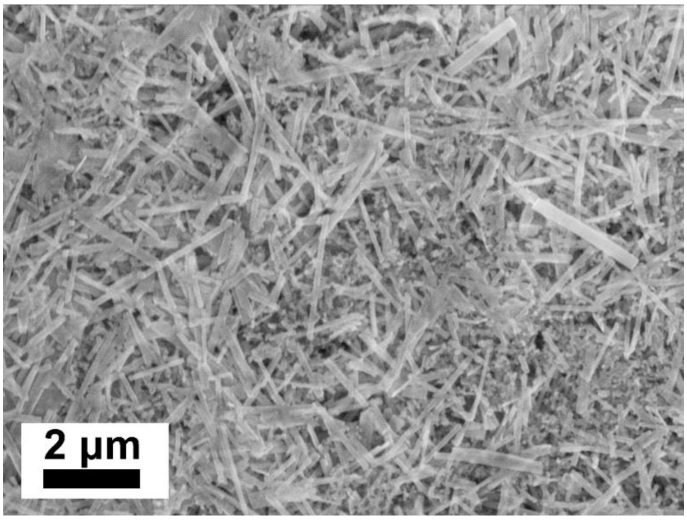

Figure S5. SEM image of $NH_4V_4O_{10}$ electrode after 1000 cycles in the voltage range of 0.2-1.6 V.

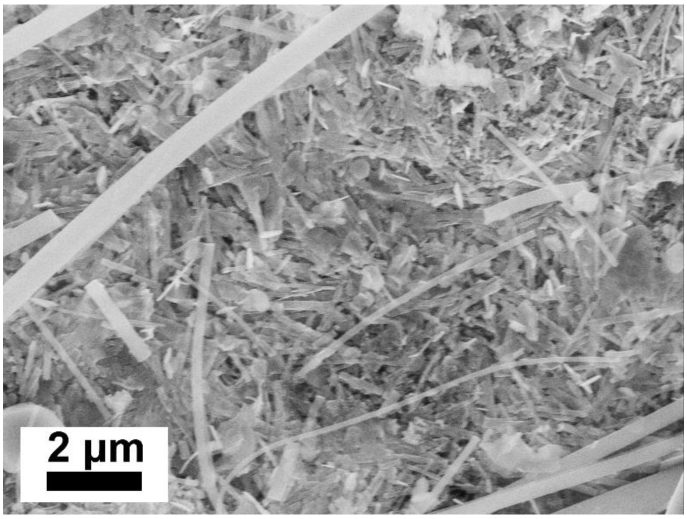

Figure S6. SEM image of $NH_4V_4O_{10}$ electrode after 1000 cycles in the voltage range of 0.2-1.4 V.

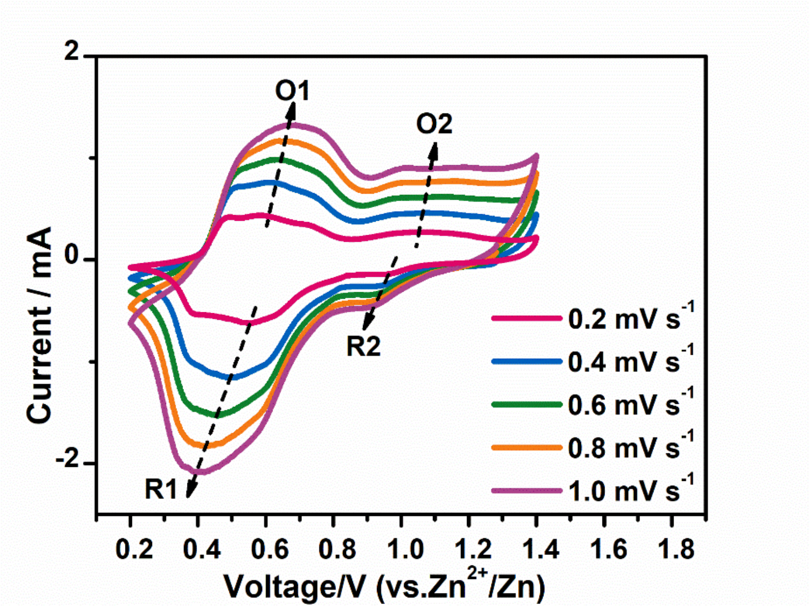

Figure S7. The CV curves of $NH_4V_4O_{10}$ at different scan rates from 0.2 to 1.0 mV s^{-1} in the voltage range of 0.2-1.4 V.

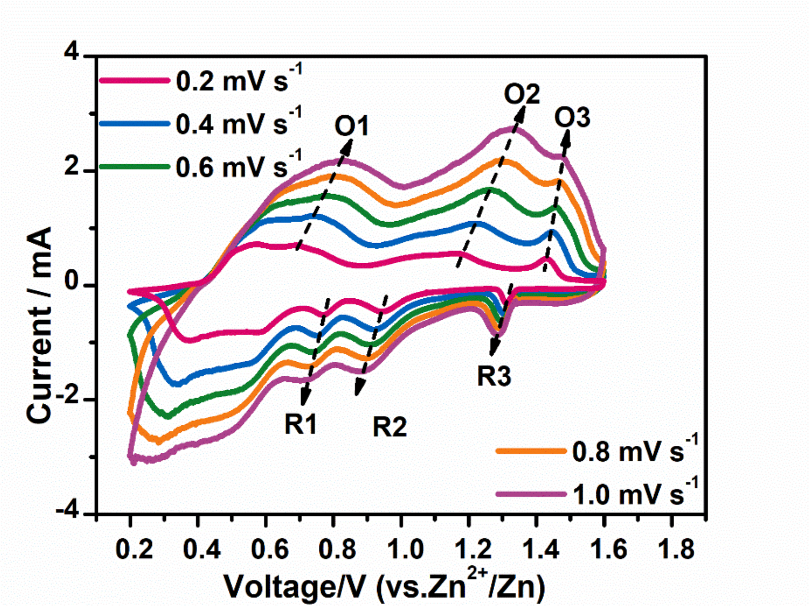

Figure S8. The CV curves of $NH_4V_4O_{10}$ at different scan rates from 0.2 to 1.0 mV s^{-1} in the voltage range of 0.2-1.6 V.

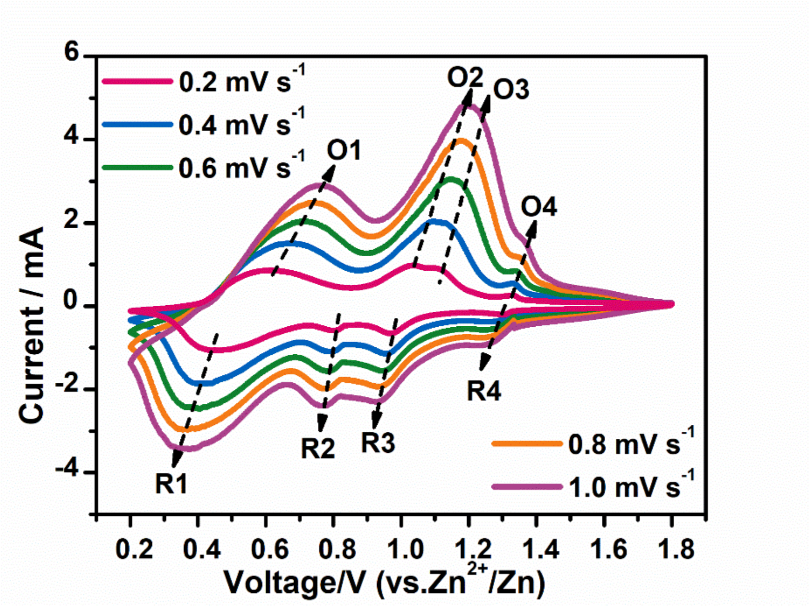

Figure S9. The CV curves of $NH_4V_4O_{10}$ at different scan rates from 0.2 to 1.0 mV s^{-1} in the voltage range of 0.2-1.8 V.

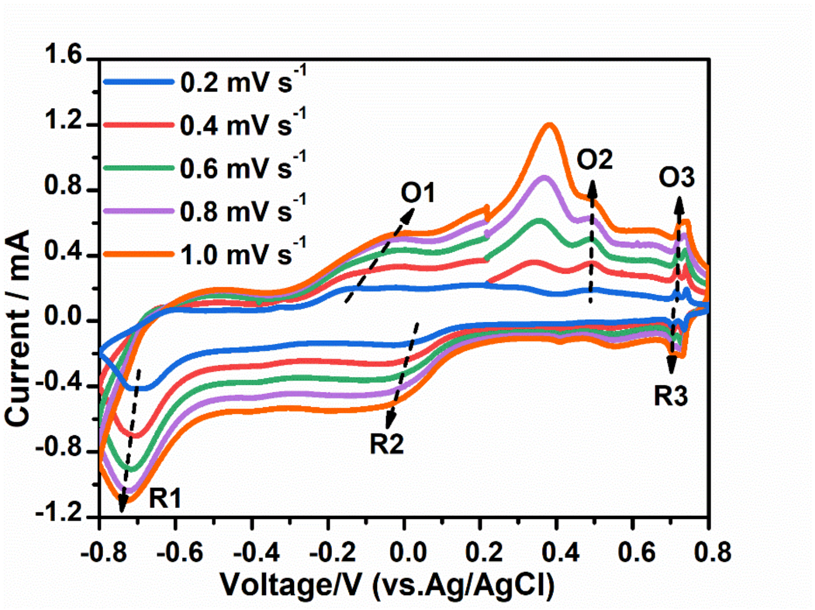

Figure S10. The CV curves of $NH_4V_4O_{10}$ at different scan rates from 0.2 to 1.0 mV s^{-1} for NH_4^+ storage.

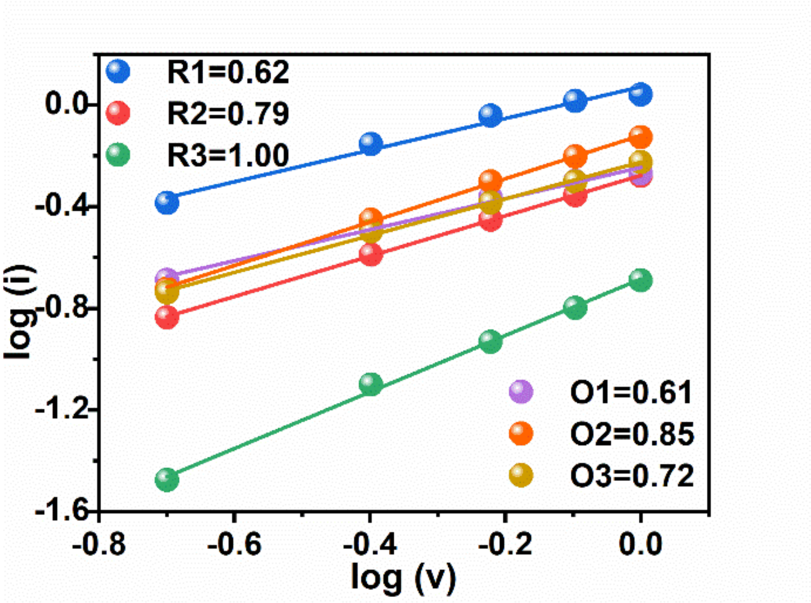

Figure S11. The *b*-values of $NH_4V_4O_{10}$ electrode for NH_4^+ storage.

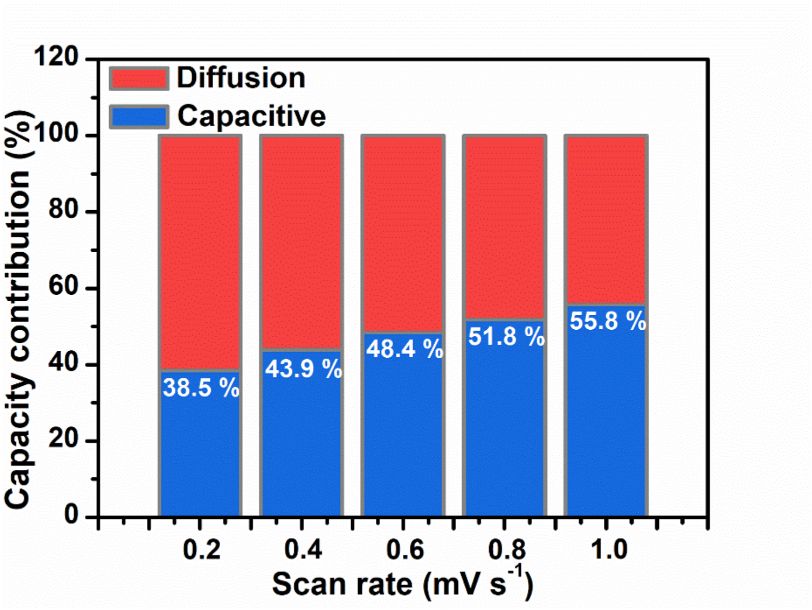

Figure S12. The capacity contribution of $NH_4V_4O_{10}$ at different scan rates for NH_4^+ storage.

Table S1. The spectra of $NH_4V_4O_{10}$ at different charge of state.

**Charge of state**	**R_s (Ω)**	**R_{ct} (Ω)**
$NH_4V_4O_{10}$-C1.4	1.09	35.81
$NH_4V_4O_{10}$-C1.6	0.74	33.87
$NH_4V_4O_{10}$-C1.8	0.58	26.16

Table S2. Electrochemical performance comparison between reported cathode materials and the $NH_4V_4O_{10}$ in the present work for NH_4^+ storage.

Cathode materials	Electrolyte	Specific capacity	Rate capability	Cycling retention	Ref.
PTMA	1 M $(NH_4)_2SO_4$	80 m Ah g^{-1} at 0.5 A g^{-1}	62 m Ah g^{-1} at 10 A g^{-1}	86.4% after 10000 cycles at 5 A g^{-1}	1
VS_2	5 M $(NH_4)_2SO_4$	120 m Ah g^{-1} at 0.1 A g^{-1}	20 m Ah g^{-1} at 5 A g^{-1}	43% after 1000 cycles at 1 A g^{-1}	2
Berlin Green	0.5 M $(NH_4)_2SO_4$	90 m Ah g^{-1} at 0.1 A g^{-1}	41 m Ah g^{-1} at 100 A g^{-1}	87% after 4000 cycles at 1 A g^{-1}	3
MoO_3	1 M NH_4Cl	115 m Ah g^{-1} at 0.1 A g^{-1}	32 m Ah g^{-1} at 15 A g^{-1}	94% after 100000 cycles at 15 A g^{-1}	4
MnO_x	0.5 M NH_4Ac	176 m Ah g^{-1} at 0.5 A g^{-1}	66 m Ah g^{-1} at 10 A g^{-1}	94.7% after 10000 cycles at 5 A g^{-1}	5
NaFeHCF	1 M $(NH_4)_2SO_4$	62 m Ah g^{-1} at 0.25 A g^{-1}	48 m Ah g^{-1} at 2 A g^{-1}	109.7% after 50000 cycles at 2 A g^{-1}	6
MnAl-LDH	0.5 M $(NH_4)_2SO_4$	184 m Ah g^{-1} at 0.1 A g^{-1}	70 m Ah g^{-1} at 5 A g^{-1}	81% after 400 cycles at 0.1 A g^{-1}	7
$NH_4V_4O_{10}$ (This work)	5 M $(NH_4)_2SO_4$	305 m Ah g^{-1} at 0.1 A g^{-1}	90 m Ah g^{-1} at 5 A g^{-1}	92.8% after 3000 cycles at 5 A g^{-1}	

References

[1] Y. Zhang, Y. An, B. Yin, J. Jiang, S. Dong, H. Dou, X. Zhang, *J. Mater. Chem. A* **2019**, 7, 11314.

[2] D. Yu, Z. Wei, X. Zhang, Y. Zeng, C. Wang, G. Chen, Z. X. Shen, F. Du, *Adv. Funct. Mater.* **2020**, 31, 2008743.

[3] X. Wu, Y. Xu, H. Jiang, Z. Wei, J. J. Hong, A. S. Hernandez, F. Du, X. Ji, *ACS Appl. Energy Mater.* **2018**, 1, 3077.

[4] G. Liang, Y. Wang, Z. Huang, F. Mo, X. Li, Q. Yang, D. Wang, H. Li, S. Chen, C. Zhi, *Adv. Mater.* **2020**, 32, 1907802.

[5] Y. Song, Q. Pan, H. Lv, D. Yang, Z. Qin, M. Y. Zhang, X. Sun, X. X. Liu, *Angew Chem. Int. Ed.* **2021**, 60, 5718.

[6] C. Li, W. Yan, S. Liang, P. Wang, J. Wang, L. Fu, Y. Zhu, Y. Chen, Y. Wu, W. Huang, *Nanoscale Horiz.* **2019**, 4, 991.

[7] Q. Liu, F. Ye, K. Guan, Y. Yang, H. Dong, Y. Wu, Z. Tang, L. Hu, *Adv. Energy Mater.* **2022**, 2202908.
